# The Secret of Secrets: Carbonic Anhydrase Concentration in Lizards' Femoral Gland Secretions Is Tuned to Environmental Conditions

**DOI:** 10.1002/ece3.72023

**Published:** 2025-08-21

**Authors:** Marco Mangiacotti, Marco Fumagalli, Gregorio Moreno‐Rueda, Francisco J. Zamora‐Camacho, José Martìn, Roberto Sacchi

**Affiliations:** ^1^ Department of Earth and Environmental Sciences University of Pavia, Università degli Studi di Pavia Pavia Italy; ^2^ Departamento de Zoología, Facultad de Ciencias Universidad de Granada Granada Spain; ^3^ Departamento de Biología de Organismos y Sistemas Universidad de Oviedo Oviedo Spain; ^4^ Departamento de Ecología Evolutiva Museo Nacional de Ciencias Naturales, CSIC Madrid Spain

**Keywords:** chemical communication, environmental factors, enzyme, homeostasis, scent marking

## Abstract

Among the modalities of animal communication, the chemical channel is the only one that allows signalers and receivers to communicate without being necessarily in the same place at the same time. This asynchrony may influence signal design, as its effectiveness depends on adapting to predictable environmental conditions. Many lizard species scent‐mark territories by depositing waxy secretions made of protein‐lipid mixtures from specialized epidermal glands. These secretions, once left on a substrate, face environmental fluctuations, and their lifespan depends on the mixture's ability to tolerate such changes. Since some proteins in these secretions are enzymes, we hypothesize they may serve homeostatic functions, enabling the mixture to actively respond to external conditions. Accordingly, we can expect that (1) enzymes remain active in secretions; and (2) their abundance varies across an environmental gradient, tuning the homeostatic ability to the predictable external conditions. We tested these predictions using carbonic anhydrase (CA), an enzyme found in many lizard secretions that regulates pH by catalyzing the reaction between water and carbon dioxide. Protonography confirmed CA activity in proteins extracted from femoral gland secretions from eight *Podarcis* lizard species and *Psammodromus algirus*. We also analyzed CA abundance in males (*N* = 70) from 12 *Ps. algirus* populations, and its correlation with the environmental gradient (geography, topography, climate). Model comparison revealed a credible relationship between CA concentration and geography as well as bioclimatic variables in *Ps. algirus*. Taken together, these findings suggest CA plays an enzymatic function that may help stabilize the internal chemical environment of secretions, potentially enhancing the scent‐mark's lifespan and effectiveness under varying environmental conditions.

## Graphical abstract

Lizard scent‐marks bear proteins known to work as enzymes, like Carbonic anhydrase, which has been hypothesized to play a homeostatic role by stabilizing the secretion's internal environment. We found that CA concentration varies according to climate‐related gradients in *Psammodromus algirus*, supporting the idea that CA helps maintain the effectiveness and lifespan of the scent marks under predictable changes in environmental conditions.

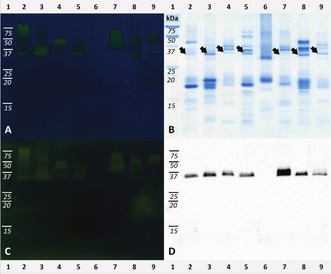

## 1 Introduction

Animal communication can exploit many different modalities (v. gr. mechanical, acoustic, chemical, visual, or electrical), each characterized by specific properties and therefore suited for specific contexts (Bradbury and Vehrencamp 2011). Within this diversity, chemical communication is considered the most ancient and bears the very peculiar property of enabling “asynchronous” communication (Wyatt 2003; Bradbury and Vehrencamp 2011). Chemical signals, in fact, allow signalers and receivers not to necessarily be in the same place at the same time for communication to take place (Nehring et al. 2013). For example, under territorial context, a territory owner leaves its scent‐mark signature on some points within its range, which can then be detected by conspecifics, obtaining information about the owner (even without it being present; Johnson 1973). Asynchronous chemical communication is common in the animal kingdom, and it is typically used to mediate intraspecific interactions like territoriality, mate choice, rival assessment, and social integration (Wyatt 2003; Bradbury and Vehrencamp 2011).

The drawback of asynchrony is that once the chemical signal has been released (e.g., as a substrate scent‐mark), it can no longer be modified by the signaler, and the scent‐mark is exposed to external factors able to alter it (Wyatt 2003; Bradbury and Vehrencamp 2011; Nehring et al. 2013). This poses constraints to signal design, whose functional effectiveness ultimately depends on how well it is adjusted to the environmental conditions where it is (usually) left (Alberts 1992; Wyatt 2010; Martín and López 2013; Apps et al. 2015). For example, fade‐out time, which is the time elapsed from deposition until complete undetectability of a signal, can be greatly affected by environmental parameters like temperature, humidity, rain, UV radiation, and wind (Alberts 1992; Martín and López 2013; Apps et al. 2015; Otte et al. 2018; Martín et al. 2024). Consequently, scent marks aimed at lasting longer have adapted their composition, favoring hydrophobic blends (Wyatt 2003; Bradbury and Vehrencamp 2011; Apps et al. 2015) and/or selecting semiochemicals with heavier molecular weights or with chemical structures able to decrease their volatility (e.g., aromatic rings, ester groups) (Alberts 1992; Wyatt 2010; Apps et al. 2015; Baeckens et al. 2018). Alternatively, other molecules may function as carriers, ligands, or fixatives of the true “smaller‐sized” semiochemical, thus slowing down its release and increasing fade‐out time (Alberts 1992; Wyatt 2010, 2014; Apps et al. 2015). The latter strategy would benefit from the use of supporting molecules in chemical mixtures, with the aim of stabilizing the blend, not only by prolonging fade‐out time, but also protecting the active compounds from exogenous factors able to degrade them (e.g., antioxidant, anti‐UV function) (Mangiacotti et al. 2023a).

Lizards are good model species to investigate the design of chemical signals in general (Baeckens 2019), and notably the use of supporting molecules (Mangiacotti et al. 2023a). Like all squamate reptiles, lizards are strongly chemically oriented and typically use tongue‐flicking behavior combined with vomerolfaction to explore the chemical environment (Schwenk 1995). They also scent‐mark the occupied areas using ad hoc secretions that are left on the substrate during locomotion or active rubbing (Mayerl et al. 2015; Martín and López 2024). The chemical signature of these scentmarks can be detected and recognized by conspecifics, which can ultimately use the information gained to mediate intraspecific interactions (Martín and López 2014, 2024). In most species, the cloacal or femoral regions bear specialized epidermal glands responsible for such secretions (Cole 1966; Mayerl et al. 2015; García‐Roa et al. 2017), which consist of waxy mixtures of lipids and proteins in variable proportions (Alberts 1990; Escobar et al. 2001; Mangiacotti, Pezzi, et al. 2019). Besides molecules active in communication, the blend hosts a set of compounds which may help to preserve the semiochemicals from degradation or fading (e.g., antioxidants, lipid‐binding molecules, anti‐bacterial) (Wyatt 2010; Tellkamp et al. 2020; Ibáñez et al. 2022). More interestingly, some of the identified proteins are enzymes known to play catalytic functions in other tissues, cell compartments, or even secretions (Tellkamp et al. 2020; Ibáñez et al. 2022; Mangiacotti et al. 2023b, 2023a, 2023). The occurrence of enzymes in the mixture has led to the hypothesis that such proteins may contribute to the system homeostasis, dynamically controlling or adjusting the internal environment according to the prevailing external conditions, and ultimately prolonging the persistency and efficacy of said semiochemicals (Mangiacotti et al. 2023). At least two predictions easily follow the homeostatic‐function hypothesis: (1) enzymes must keep their catalytic activity in the secretions; and (2) the enzyme concentrations should vary according to some environmental features of the site where they are usually released. The former is a necessary condition to support the homeostatic‐function hypothesis; the latter stems from the consideration that if enzyme occurrence is functional to the chemical stability of the mixture, its abundance should be calibrated on the predictable and repeatable characteristics of the external environment. A similar tuning has been observed for other components of the secretions, whose concentrations respond to different environmental gradients (e.g., thermal, pluviometric, elevational; Khannoon et al. 2013; Martín et al. 2017; Baeckens et al. 2018).

In order to verify the homeostatic‐function hypothesis, we tested the above predictions in a well‐studied lizard group, lacertids, and on a specific enzyme, carbonic anhydrase (CA). Lacertidae is an Old‐World squamate family, which has been the focus of many studies on chemical communication in the last 40 years (Baeckens 2019). Adult males typically bear two series of epidermal glands along the thighs (femoral glands, FG) whose waxy secretions are used in scent marking (Baeckens et al. 2015; Mayerl et al. 2015; Martín and López 2024). A comprehensive list of the composition of the proteinaceous fraction is available for only two lizard species, the lacertid *Lacerta agilis* (Ibáñez et al. 2022) and the iguanid *Amblyrhynchus cristatus* (Tellkamp et al. 2020). In both species, CA is cataloged among the identified enzymes, and, due to its hydratase activity catalyzing the reversible hydration of carbonic dioxide into carbonic acid, it was thought to contribute to the pH regulation of the chemical matrix (Tellkamp et al. 2020; Ibáñez et al. 2022). The occurrence of carbonic anhydrase in the secretions was later demonstrated in other lacertids, and the enzyme was shown to be evenly distributed within the mixture (Mangiacotti et al. 2023). CA abundance was also found to correlate with the composition of the lipophilic fraction (Mangiacotti et al. 2023a, 2023b), notably with some specific compounds known to be active in communication (like provitamin D_3_) and which can be highly sensitive to any variation in the chemical environment. The catalytic activity of CA may therefore contribute to stabilizing the chemical matrix, thus possibly prolonging the lifespan and efficiency of such molecules. We thus prepared and analyzed two sets of FG secretion samples available from previous studies (Table 1). The first set included samples from eight congeneric lacertid species, genus *Podarcis*, for which a previous study validated the occurrence of CA in FG secretions (Mangiacotti et al. 2023). This set was primarily used to test CA activity (prediction #1) and was therefore subject to protonography, a technique specifically designed to detect hydratase activities (De Luca et al. 2015). The second set was from 12 Spanish populations of the large psammodromus (*Psammodromus algirus*) collected across a geographical and elevational gradient (Martín et al. 2016, 2017). After demonstrating that CA is present and functionally active in the secretions of this species, we used *Ps. algirus* samples to test the occurrence of any relation between CA concentration and environmental gradients (prediction #2).

**TABLE 1:** Synthesis of information about the samples used in the present study. For each species, we reported: Collection country (Country) and geographic coordinates (Longitude E, Latitude N); year of collection (Year); number of samples used in this study (N); the analysis the sample was used for (Use: Environmental gradient, Protonography, or Mass Spectrometry, MS); the reference paper (Reference).

Species	Country	Geographic coordinates		Year	*N*	Use	References
		Longitude E	Latitude N				
*Podarcis bocagei*	Spain	−9.08	42.74	2007	1	Protonography	Mangiacotti et al. (2023)
*Podarcis carbonelli*	Spain	−6.09	40.50	2007	1	Protonography	
*Podarcis erhardii*	Greece	24.85	37.83	2014	1	Protonography	
*Podarcis gaigae*	Greece	24.25	38.95	2014	1	Protonography	
*Podarcis liolepis*	Spain	1.84	42.75	2012	1	Protonography	
*Podarcis melisellensis*	Croatia	14.34	45.10	2013	1	Protonography	
*Podarcis milensis*	Greece	24.44	36.69	2015	1	Protonography	
*Podarcis muralis*	Italy	9.23	45.24	2011	1	Protonography	
*Psammodromus algirus*	Spain	−4.62	40.17	2014	5	Gradient	Martín et al. (2016)
		−4.03	40.72	2014	12	Gradient/Protonography/MS	
		−3.94	40.22	2014	4	Gradient	
		−3.75	40.51	2014	4	Gradient	
		−2.60	40.04	2014	9	Gradient/Protonography	
		−3.59	40.02	2014	4	Gradient	
		−3.32	37.03	2013	5	Gradient	Martín et al. (2017)
		−3.42	36.92	2013	6	Gradient	
		−3.44	36.92	2013	5	Gradient	
		−3.41	36.89	2013	6	Gradient/Protonography/MS	
		−3.43	36.95	2013	4	Gradient	
		−3.32	36.97	2013	6	Gradient/Protonography/MS	

## 2 Material and Methods

### 2.1 Sampling Femoral Gland Secretions

**FIGURE 1:**
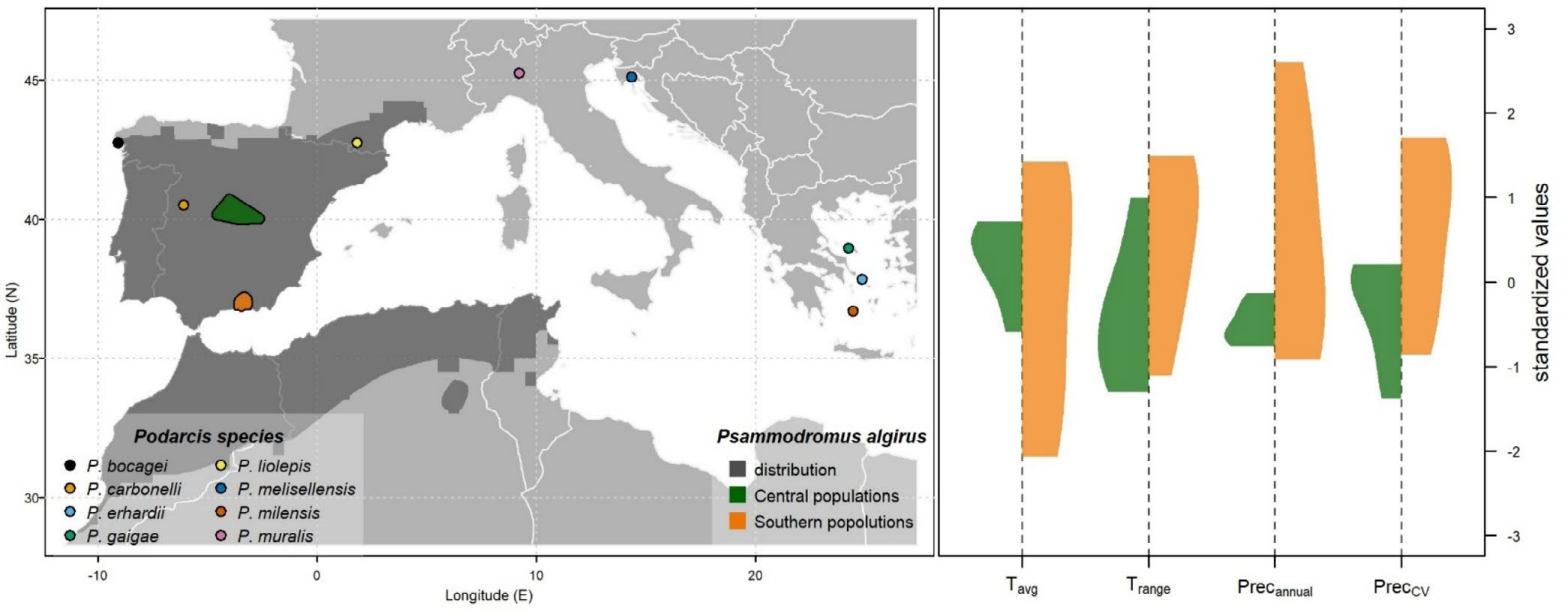
Distribution of the sampling localities of the lizard species used in the study. *Left panel*: Dots mark the sampling site for the eight *Podarcis* species; green and orange polygons define the two geographic regions (central and southern Spain, respectively) from which *Psammodromus algirus* populations were sampled; dark‐gray shaded area represents the distribution of *P. algirus* (Roll et al. 2017). *Right panel*: Violin plot of climatic variability across the two geographic regions of *P. algirus* populations; *T*_avg_ = average annual temperature; *T*_range_ = temperature mean diurnal range; Prec_annual_ = total annual precipitation; Prec_CV_ = Precipitation seasonality (coefficient of variation); all variables were standardized before plotting to allow comparing different scales.

Samples of FG secretions used in this study were collected from 2007 to 2015 during field activities related to other studies (Table 1; Figure 1, left panel; Martín et al. 2016, 2017; Mangiacotti et al. 2023). Collection procedures were not invasive and did not cause damage to animals (Fitzgerald 2012; Baeckens et al. 2017). We used glass vials with glass inserts to collect directly FG secretions that had been extracted by gently pressing around the femoral pores. We closed vials with Teflon‐lined stoppers and stored them at −20°C until analysis. All lizards were released healthy at their capture points after sample collection. Previous studies about protein from femoral gland secretions had demonstrated that the observed timelapse from collection to analysis did not bias electrophoretic and mass spectrometry analysis (Mangiacotti et al. 2017, 2021; Mangiacotti et al. 2023a, 2023b; Mangiacotti et al. 2023). This study complies with the ARRIVE guidelines (du Sert, Hurst, et al. 2020; du Sert, Ahluwalia, et al. 2020).

### 2.2 Testing CA Activity: Protonography

To assess whether CA was still active in the secretions, we used protonography, a technique specifically designed for the detection of this enzyme class in SDS‐PAGE, based on the pH variation associated with the catalytic conversion of CO_2_ into HCO_3_^−^ and H^+^ (De Luca et al. 2015). We applied protonography to the same samples (Table 1) used in a previous work validating CA identification in FG secretions in eight *Podarcis* species (two males per species) employing immunoblotting and immunocytochemical techniques (Mangiacotti et al. 2023). Here, we used a single individual for each of the eight *Podarcis* species. Furthermore, we applied protonography to four randomly chosen samples (from different populations) from the *Psammodromus algirus* group in order to confirm CA activity also in this species.

Protonography was performed following the procedure outlined in De Luca et al. (2015). Individual samples were prepared by adding 10 μL of loading buffer solution (50 mM Tris–HCl pH 6.8, 2% sodium dodecyl sulphate, 0.1% bromophenol blue, and 10% glycerol) to aliquots of 10 μg of proteins for each sample. The boiling step was avoided to prevent protein denaturation, and electrophoresis was run in a discontinuous mode (5% stacking gel and 15% running gel) at 180 V constant voltage for about 2 h. The obtained gel was dipped in 2.5% Triton X‐100 for 1 h and then twice in 100 mM Tris, pH 8.2, containing 10% isopropanol for 10 min. Finally, the gel was immersed in 0.1% bromothymol blue in 100 mM Tris, pH 8.2 for 30 min. To assess any hydratase activity, the gel was soaked in CO_2_‐saturated ddH_2_O, and the enzymatic reaction was marked by the local pH change, from alkaline to acidic, made visible by the indicator turning from blue to yellow (De Luca et al. 2015). A gel image was acquired as the color change started. The gel was then immersed in the fixing solution (acetic acid 10% v/v and methanol 40% v/v) for 1 h to remove the bromothymol blue solution and fix protein positions. Then the gel was stained with Coomassie Blue G‐250 to obtain the corresponding banding scheme.

### 2.3 Testing CA Concentration Variability: Environmental Gradient Effect

FG secretion samples used in this analysis came from previous studies that investigated the variability of the lipophilic fraction in 12 Spanish populations of *Psammodromus algirus* (Martín et al. 2016, 2017). Populations were distributed across an elevational gradient (from 300 to 2500 m a.s.l.) and a geographical gradient (Figure 1, Table 1). FG secretions from 4 to 12 males for each population (Table 1) were defatted and prepared for sodium dodecyl sulphate‐polyacrylamide gel electrophoresis (SDS‐PAGE) according to well‐established protocols described in previous studies (Mangiacotti et al. 2017, 2021; Mangiacotti, Pezzi, et al. 2019; Mangiacotti, Fumagalli, et al. 2019). Aliquots containing around 10 μg of proteins for each sample were used in each electrophoretic run (discontinuous mode; 5% stacking gel and 15% running gel, constant voltage of 180 V for 2 h), and the obtained gels were stained with Coomassie Blue G‐250 solution. After destaining with acetic acid 5% (v/v) solution, high‐quality images of the gels were acquired. Gel images were aligned to obtain normalized electrophoretograms for each lane (Mangiacotti, Pezzi, et al. 2019; Mangiacotti et al. 2021) and the band putatively corresponding to CA was identified based on its position (molecular weight) along the lane and its univocal spatial relations with the nearby peaks in the electrophoretogram (Figure S1; Mangiacotti et al. 2023). We measured the area under the CA peak and used it as a proxy for the relative abundance of the enzyme in the secretion (hereafter CA concentration; Figure S1; Candiano et al. 2004; Yrjänäinen et al. 2022).

To validate CA identification, we randomly chose three FG secretion samples, performed an electrophoretic run, excised the three CA bands from the obtained gel (Figure S2), and analyzed them with tandem Mass Spectrometry (MS). We prepared samples for MS following Mangiacotti et al. (2023): gel pieces were physically fragmented, destained, reduced, dehydrated, and then digested with sequencing grade trypsin (Promega). The resultant peptides were extracted from the gel matrix and solubilized in a 0.1% (v/v) formic acid water solution. MS analyses were carried out with a high resolution QTOF mass spectrometer (X500B, AB Sciex). Peptides were separated by reverse phase (RP) HPLC on a Hypersil Gold (Thermo Fisher Scientific) C18 column (150 × 2.1 mm, 3 μm particle size, 175 Å pore size) using a linear gradient (2%–50% solvent B in 15 min) in which solvent A consisted of 0.1% aqueous formic acid and solvent B of acetonitrile containing 0.1% formic acid. Flow rate was 0.2 mL/min. Mass spectra were generated in positive polarity under constant instrumental conditions (ion spray voltage: 4500 V; declustering potential: 100 V; curtain gas: 30 psi; ion source gas 1 40 psi; ion source gas 2 45 psi; temperature: 350°C; collision energy 10 V). Protein identification was performed using peptide‐spectrum matching (Eng et al. 2011) in MS‐GF+ v2014.03.26 (Kim and Pevzner 2014) with the following settings: tolerance, 30 ppm; charge range, 1–6+; range of peptide length, 7–70; isotope error 0–1 Da; cleavage, tryptic; post translational modification, fix carbamidomethylation of cysteine (Mangiacotti, Fumagalli, et al. 2019). Since no specific proteome was available for *Ps. algirus*, three *ad hoc* databases were considered: the list of 19,107 protein sequences available in UniProtKB release 2024_05 (The UniProt Consortium 2023), searched for Carbonic Anhydrase and filtered for vertebrates; the available proteomes of phylogenetically related lizards (con‐familiar) *Podarcis muralis* (UP000472272) and *P. lilfordi* (UP001178461) (The UniProt Consortium 2023). Peptide‐spectrum matching was run using a target‐decoy approach and identification was scored according to the spectrum E‐value and false discovery rate (Everett et al. 2010; Bern and Kil 2011; Jeong et al. 2012). Only fully tryptic peptides with false discovery rate smaller than 0.01 were considered (Mangiacotti, Fumagalli, et al. 2019). Additionally, four randomly chosen samples were also used in protonography to assess CA activity (see previous section).

In order to investigate the relationship between CA concentration and environmental gradient, we used linear mixed models fitted under a Bayesian framework through the R package “brms” v2.21.0 (Bürkner 2017). A model selection approach was adopted, where CA concentration was always included as the response variable, the population of origin as the random intercept, but different predictors were used as environmental proxies. A total of four competing models were eventually built: two based on bioclimatic variables (*bioclimatic* models); one based on altitude alone (*topographic* model); and one based on the geographic region (Central or Southern Spain) the site belonged to (*geographic* model). The first two models aimed to represent a more explicit hypothesis concerning the potential relation between thermal and pluviometric conditions and CA abundance. For each site, we indeed extracted the values of four bioclimatic variables commonly used to describe macroclimate (Figure 1; Fick and Hijmans 2017): average annual temperature (mean of the monthly mean temperatures; *T*_avg_); mean diurnal range (mean of monthly difference between the maximum and minimum temperature; *T*_range_); annual precipitation (the cumulated monthly rainfall over a year; Prec_annual_); precipitation seasonality (coefficient of variation of the monthly rainfall over a year; Prec_CV_). The bioclimatic variables were calculated from the monthly values of mean, minimum, and maximum temperature and total rainfall as computed by ClimateEU v4.63 (Marchi et al. 2020) over the 30‐year interval 1991–2020. The information about elevation needed to perform downscaling was extracted from the digital elevation model of Spain at 200 m spatial resolution (https://centrodedescargas.cnig.es/CentroDescargas/busquedaSerie.do?codSerie=MDS05). Since bioclimatic variables showed high correlations and collinearity (Table S1), from the four original variables we obtained two subsets of three not‐collinear variables (Table S1) that we used to build the corresponding bioclimatic models: *bioclimatic1* using *T*_avg_, *T*_range_, and Prec_CV_ as fixed effect, and *bioclimatic2* using *T*_range_, Prec_annual_, and Prec_CV_. All predictors were standardized to zero mean and unit variance by subtracting the mean and dividing by the standard deviation (scale function in the basic R stat). Models were run with default brms settings (chains = 4; iter = 2000, warm‐up = 1000, thin = 1) (Bürkner 2017) and convergence was checked. Draws from the posterior distribution were reported as MAP (Maximum A Posteriori probability estimate) and 50% and 95% highest density intervals (HDI_50_; HDI_95_) (Makowski et al. 2019). The existence of the effects of a parameter was based on MPE (Maximum Probability of Effects), the probability of a coefficient being positive or negative (Makowski et al. 2019). We performed model comparison in the Bayesian framework using expected long pointwise predictive density (*elpd*) calculated via leave‐one‐out cross‐validation (Vehtari et al. 2017) and implemented in the loo package v2.7.0 (Vehtari et al. 2024). Models were ranked by *elpd* scores (the higher, the better), and a model was considered less informative than the best one if its relative difference (Δ*elpd*) plus the associated standard error (SE_Δ*elpd*_) did not include zero.

## 3 Results

### 3.1 Testing CA Activity: Protonography

**FIGURE 2:**
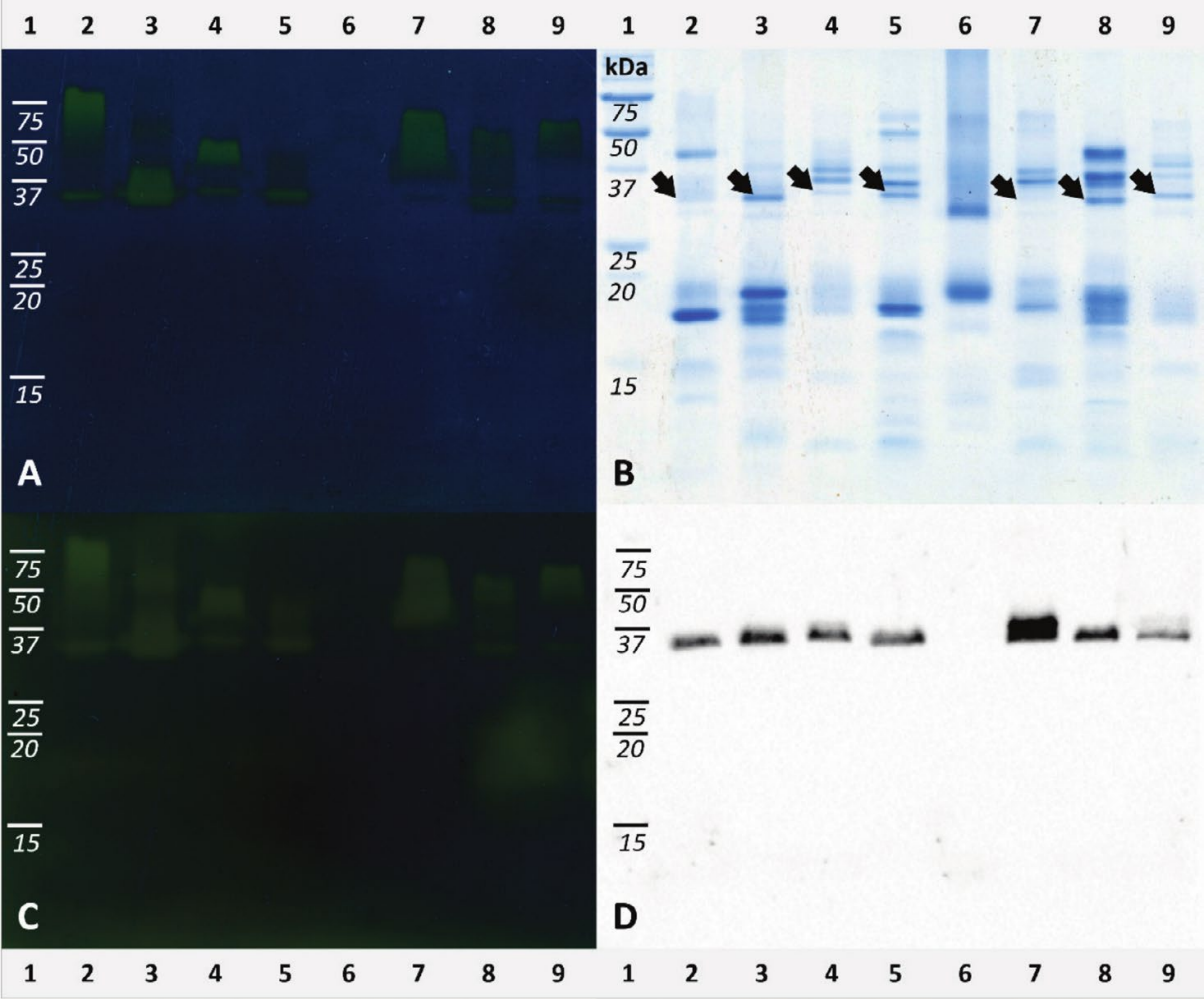
Protonography of *Podarcis* samples compared to SDS‐PAGE and western blot. (A) Protonography (10 s incubation time). (B) SDS‐PAGE stained with Coomassie blue after protonography. (C) Protonography (20 s incubation time). (D) Western blot of the same samples (modified from figure 5 in Mangiacotti et al. (2023)). Lanes numbering is reported above and below gel images. Samples were always loaded in the same order: 1 = molecular weights (kDa); 2 = *P. gaigae*; 3 = *P. erhardii*; 4 = *P. bocagei* ; 5 = *P. melisellensis*; 6 = *P. liolepis*; 7 = *P. carbonelli* ; 8 = *P. milensis*; 9 = *P. muralis* . Black arrows mark the position of the CA band in SDS‐PAGE lanes. Gel A, B, and C were cropped and color‐balanced in Adobe Photoshop CS3. Original images are available as (Figure S3).

In the protonography of the eight *Podarcis* lizards, a well‐defined yellow‐greenish band just below 37 kDa appeared in six out of the eight lanes after 10 s incubation (Figure 2A). The same mark also became visible in lane 7 ( *P. carbonelli* ) after 20 s incubation (Figure 2C), while it was completely missing in lane 6 (*P. liolepis*). Hydratase activity was therefore detected in all but one case. The position of the bands matched those of the CA bands in the same Coomassie‐blue stained gel (Figure 2B) and in the western blot (Figure 2D) obtained using a polyclonal antibody against vertebrate CA‐IV (Mangiacotti et al. 2023): it approximately corresponded to the predicted molecular weight of *P. muralis* CA‐IV (about 35.9 kDa; Mangiacotti et al. 2023). Neither Coomassie‐stained gel nor western blot presented a matchable band for lane 6, confirming the lack of reaction in protonography for *Podarcis liolepis*. Further, all the samples positive for hydratase activity showed an additional yellowish region above 37 kDa, which appeared scattered and less defined compared to the CA‐matching one (Figure 2A,C).

**FIGURE 3:**
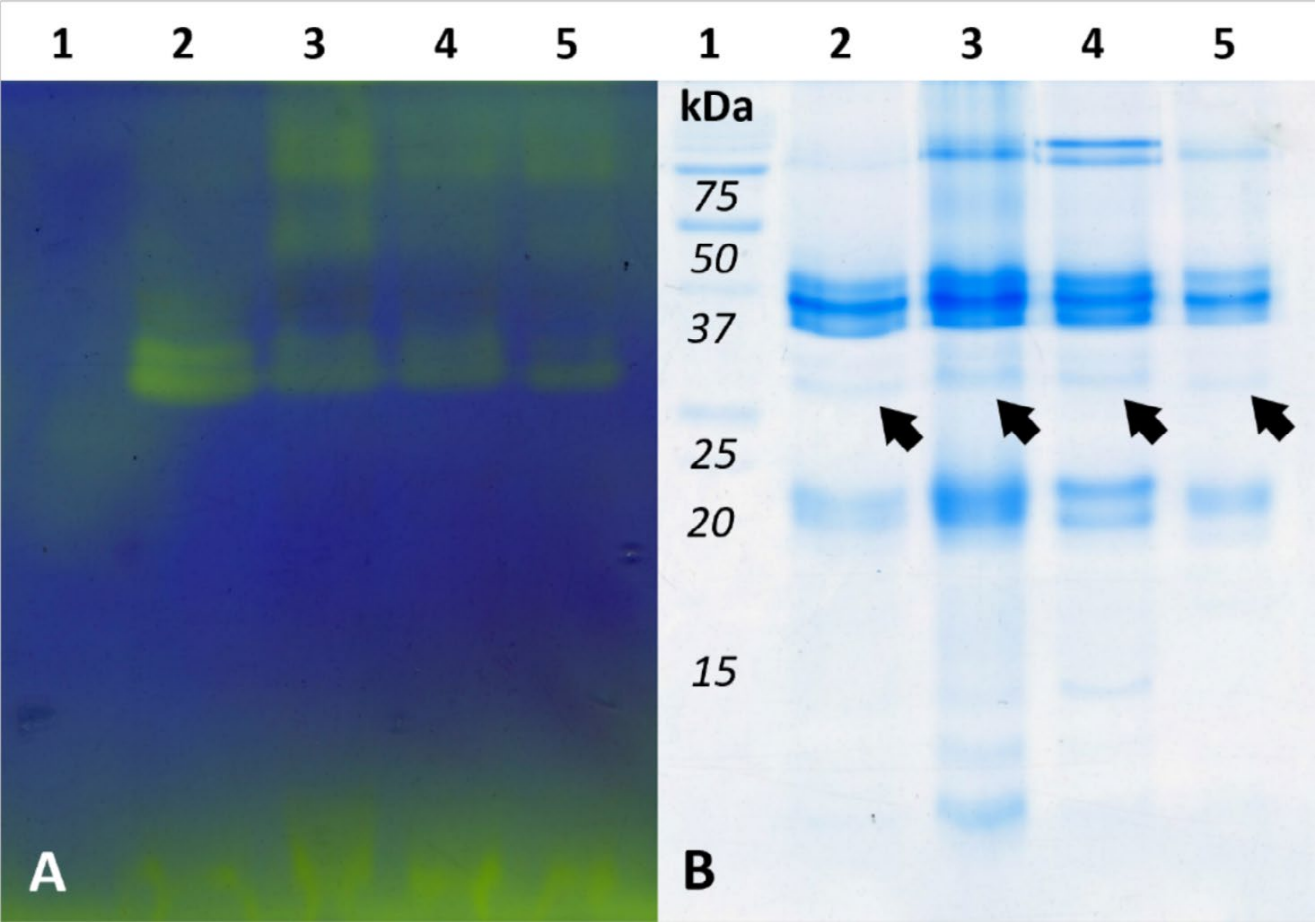
Protonography of *Psammodromus algirus* samples compared to SDS‐PAGE. (A) Protonography (20 s incubation time). (B) SDS‐PAGE stained with Coomassie blue after protonography. Samples were loaded on the same lane in each gel, from lane 2 to lane 5; lane 1 loaded molecular weights. Black arrows mark the position of the CA band in SDS‐PAGE lanes. Gel A and B were cropped to the zone of interest and brightness enhanced to improve visualization in Adobe Photoshop CS3. Original images are available as (Figure S4).

All the four samples of *Psammodromus algirus* used in protonography showed yellowish well‐defined bands (Figure 3A) corresponding to CA bands identified by MS (Figure 3B; Table S2) and occupying a lighter molecular region compared to the *Podarcis* samples (Figures 2B and 3B). Differently from the previous protonography, *Ps. algirus* banding appeared double, with a less evident thin band just above the main one (Figure 3A). As with *Podarcis* spp., on the other hand, a color change also occurred in the region above 37 kDa in three out of four samples (Figure 3A): the pattern was less smeared than the one observed in *Podarcis*, albeit not easily attributable to the visible bands in the same region of the Coomassie‐blue stained gel (Figure 3B).

### 3.2 Testing CA Abundance Variability: Environmental Gradient Effect

A total of 70 FG secretions samples were used for the analysis of the environmental gradient (Table 1), and they all gave clearly identifiable and measurable CA peaks. CA concentration (relative area under the electrophoretogram; see methods) was found to be on average 1.93%, ranging from 0.97% to 3.57%. Mass Spectrometry confirmed that the peaks identified in the gel actually corresponded to CA as the only protein in the excised bands, and results were coherent among databases (Table S2): the database entries corresponding to carbonic anhydrase were the only ones showing multiple matches and scoring high (6–7 order of magnitude larger than the other proposed matches), thus indicating that different peptides from the same protein were identified in the analyzed samples, and making protein identification reliabl.

**FIGURE 4:**
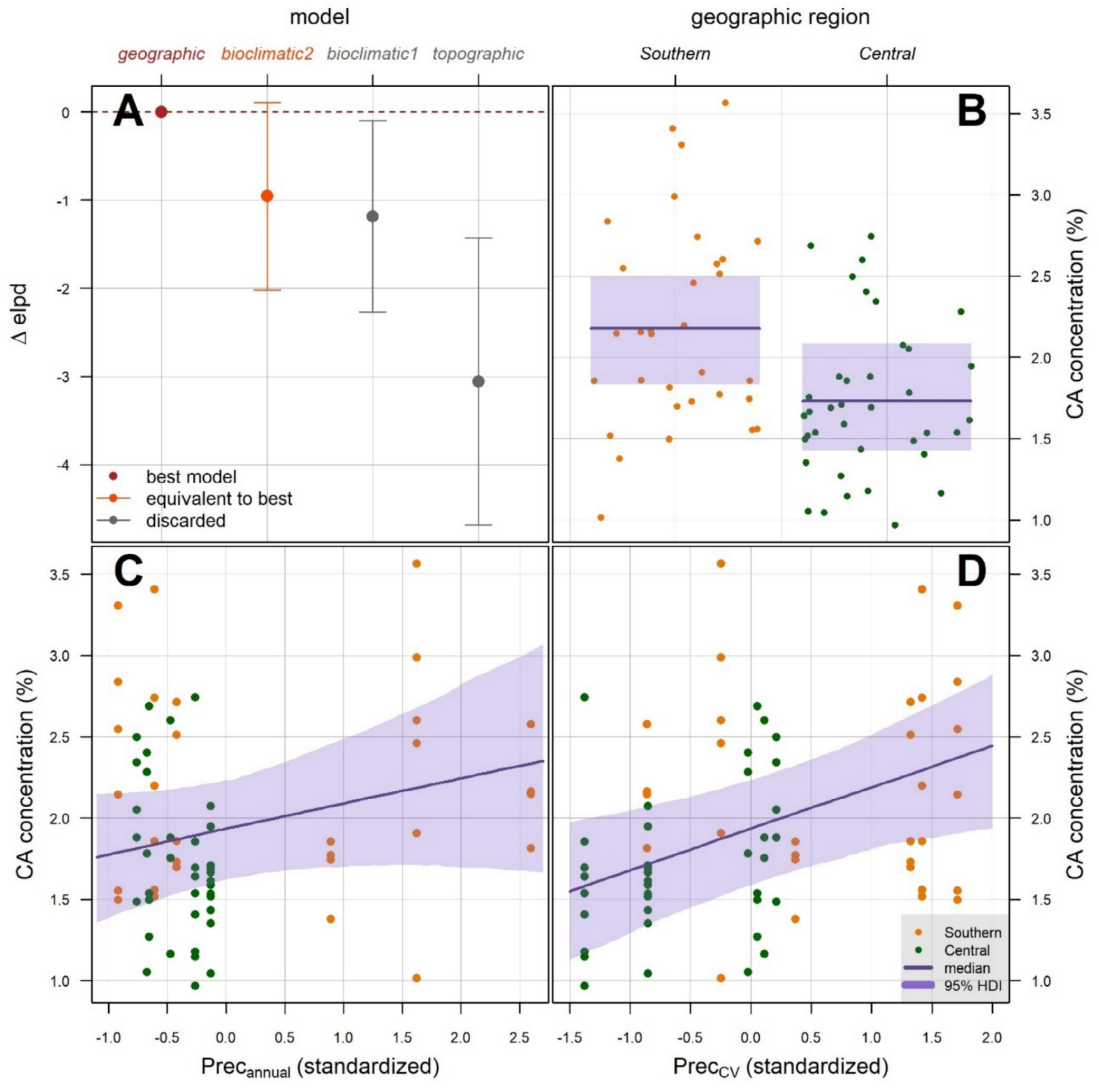
Results from the linear mixed model of the gradient analysis. (A) Model comparison according to the expected long pointwise predictive density (*elpd*): Points represent *elpd* difference with the best model (Δelpd), vertical segments represent the one‐standard‐error interval around the mean difference. (B) posterior prediction of the effect of geographic region on CA concentration from the *geographic* model. (C) posterior prediction of the effect of total annual precipitation (Prec_annual_) on CA concentration as predicted by *bioclimatic2* model. (D) posterior prediction of the effect of precipitation seasonality (Prec_CV_) on CA concentration as predicted by *bioclimatic2* model.

Among the four linear mixed models used to analyze the relation between environmental gradient and CA amount in the secretions, the geographic model was the best one, closely followed by the two bioclimatic models, and a bit farther by the topographic model (Table 2). The difference between the geographic and bioclimatic2 model can be considered negligible, as the zero value is included within one standard error from mean Δ*elpd* (Figure 4A). Looking at the coefficient estimations of the predictors used in these two best models (Table 3), we found that: (i) populations from the central region showed a lower CA concentration than southern populations (Maximum Probability of Effect, MPE > 0.99; Table 3; Figure 4B); (ii) annual precipitation and precipitation seasonality positively affected CA concentration (MPE > 0.9 for both; Table 3; Figure 4C,D), with the effect of seasonality more apparent and supported (MPE > 0.99; Table 3). Conversely, temperature range seemed not to influence secreted CA proportion (MPE = 0.555; Table 3). Considering the coefficient estimates for the two discarded models (*bioclimatic1* and *topographic*), only precipitation seasonality from *bioclimatic1* model confirmed a highly credible positive value (MPE = 0.985; Table S3).

**TABLE 2:** Model comparison results for Bayesian linear mixed models, ranked by expected log pointwise predictive density (*elpd*) Reported values include the standard error of the *elpd* estimate (SE_*elpd*_), the difference in *elpd* relative to the best model (Δ*elpd*), and the standard error of that difference (SE_Δ*elpd*_).

model	*elpd*	SE_*elpd*_	Δ*elpd*	SE_Δ*elpd*_
*Geographic*	−59.850	5.894	0.000	0.000
*Bioclimatic2*	−60.805	6.083	−0.955	1.062
*Bioclimatic1*	−61.032	6.150	−1.183	1.085
*Topographic*	−62.908	6.394	−3.058	1.628

**TABLE 3:** Coefficient estimates for the two best models of the environmental gradient analysis. Model = model for which parameters are reported; parameter = predictor for which parameter was estimated; MAP = Most A Posteriori probability estimate; HDI_95_ = 95% high density interval; MPE = Maximum Probability of Effect, probability of the parameter being strictly positive or negative.

Model	Parameter	MAP	HDI_95_		MPE
			Lower	Upper	
Geographic	Intercept	2.167	1.967	2.409	1.000
	Region (central)	−0.447	−0.747	−0.120	0.993
Bioclimatic2	Intercept	1.931	1.783	2.099	1.000
	*T*_range_	0.010	−0.220	0.256	0.555
	Prec_annual_	0.153	−0.093	0.384	0.916
	Prec_CV_	0.269	0.079	0.443	0.994

## 4 Discussion

The homeostatic‐function hypothesis states that enzymes occurring in lizard FG secretions allow the internal environment of the secretion to keep stable chemical conditions as external factors vary; thus, prolonging signal fade‐out time and its efficacy. It follows that (1) enzymes need to be active in the secretions and (2) their relative abundance should correlate with predictable changes in environmental conditions. Our results agree with both predictions, as we found that enzyme CA is active in the secretions of the eight lacertid species where it occurs, and the enzyme concentration varies according to the geographic locations and associates with bioclimatic variables (precipitation and precipitation variability) in different populations of *Psammodromus algirus*.

Protonography applied to the whole proteinaceous content extracted from FG secretions of *Podarcis* lizards highlights the occurrence of a clear and localized color change corresponding to the CA band in Coomassie‐stained gel and immunoblotting (Figure 2). As a counterproof, the same band is completely missing in *P. liolepis*, coherently with the absence of the enzyme in the secretion of this species (Mangiacotti et al. 2023). Speculating about the possible reasons for missing CA (and other enzymes with anhydrase properties) in *P. liolepis* secretions, we can notice that the electrophoretic pattern is rather simplified compared to the other *Podarcis* species (Figure 2; see Mangiacotti et al. 2023) and it also corresponds to a less complex lipid composition (Baeckens et al. 2018; Mangiacotti et al. 2023a). The lower complexity may reduce the need for tuning components (like CA) since active molecules protected by the enzyme might no longer be present. Such conditions may be adaptive or not (Caeiro‐Dias et al. 2018), and the identification of the drivers would require a specific study, starting from sampling different populations across the species range: we do indeed acknowledge that all information about FG secretions of *P. liolepis* came from a single population sampled at high elevation in the Pyrenees (1773 m a.s.l.; Mangiacotti et al. 2023), and can therefore reflect peculiar local conditions. A certain degree of interpopulation variability has indeed been observed in the lipophilic fraction of the secretions (Ortega et al. 2019), suggesting the same could happen to the proteinaceous counterpart.

A second, less defined region above the CA band shows a color change in the protonography. Such a region may correspond to CA dimer and/or trimer (De Luca et al. 2015), although no clearly distinguishable banding scheme in the same region appears in the corresponding Coomassie‐blue stained gel, or in immunoblotting. Support for such interpretation comes from two main observations: color change occurs only in the region of heavier molecular weight than the identified CA band; the absence of the CA band in the *P. liolepis* lane corresponds to the absence of any other bands in protonography. On the other side, a lower sensitivity of the Coomassie blue coloration may explain the imperfect matching with protonography, or, alternatively, polymerization may have prevented the antibody from recognizing the epitope in immunoblotting (Jiang et al. 2004). Alternatively, other heavier enzymes with esterase activity (CA also has an esterase activity; Demir et al. 2000) can react with CO_2_ and may be responsible for the additional bands. Protonography on *Psammodromus algirus* samples shows a similar pattern as in the *Podarcis* group, with a clear and defined band at about 30 kDa corresponding to CA (Table S2; Figure S2), and other active zones in the region above. So, we can confirm that CA keeps its potential catalytic activity in secretions, verifying the first prediction.

The concentration of CA in the secretion of *P. algirus* responded to both geographic and bioclimatic factors, being higher in populations from southern Spain compared to those from central Spain, and showing a positive association with rainfall and the extent of its yearly variation. Precipitation amount and distribution can potentially affect the persistence and efficacy of chemical scent‐marks both directly, by physically acting on them (e.g., washing them out), and indirectly, by influencing microclimatic conditions such as humidity (Alberts 1992; Apps et al. 2015; Martín et al. 2017). The latter factor may alter blend pH, which may justify an increased CA concentration as a compensatory mechanism. Unfortunately, the correlative approach is not able to demonstrate any causal effect; the environmental factors included in the bioclimatic model might be a proxy for other, unmeasured variables responsible for the true causal relation. This may explain why the geographic model performed equally or better than the bioclimatic one: central and southern regions exhibit not only quite different climates (Beck et al. 2023) but also differ in geology (www.igme.es), soil (Ballabio et al. 2016), land use (Corine Land Cover 2018, European Union's Copernicus Land Monitoring Service information, https://doi.org/10.2909/960998c1‐1870‐4e82‐8051‐6485205ebbac), and consequently vegetation and substrate characteristics. All the above factors can potentially influence the chemical composition of the blend (Heathcote et al. 2014; Apps et al. 2015; Martín et al. 2017; Iglesias‐Carrasco et al. 2018; Baeckens et al. 2025), including CA concentration. The geographic model may have captured part of the variation not strictly related to the bioclimatic variables considered, providing a better correlative model. Nonetheless, despite our approach not being able to rule out one parameter in favor of the other, it still supports the prediction based on the homeostatic hypothesis that a correlation between climate (or other site conditions) and enzyme abundance should occur.

An analogous environmental correlation has been observed in the composition of the lipophilic fraction in populations sampled along an elevational gradient in *P. algirus* (Martín et al. 2017); the relative abundances of some classes of compounds, but not others, increase with altitude, in a way that the physical chemical characteristics of these compounds might optimize signal efficiency in the face of the microclimate changes with elevation (notably, humidity, UV, temperature). So, through different, not exclusive pathways, both mechanisms (one acting on the composition of the volatile fraction and one on the homeostatic properties of the mixture) apparently lead to adapting signal efficacy to the environmental context, and may explain the covariation of the lipid and protein profiles (including CA) observed in lacertids (Mangiacotti et al. 2023a). Previous studies have indeed shown how persistency and efficacy of FG secretions suffer from the thermal conditions they are exposed to, and heating reduces fade‐out time and detectability (Martín and López 2013; Martín et al. 2015). Also, an interpopulational difference in the attenuation induced by the thermal treatment has been observed in some species (Martín et al. 2015; Iglesias‐Carrasco et al. 2018), suggesting that local adaptation can occur. Finally, at least the lipophilic fraction of the FG secretion can be plastically modified according to the conditions previously experienced by the signaler (Heathcote et al. 2014), or to the interannual variation in the environmental factors (Baeckens et al. 2025). All the above considerations strengthen the idea that FG secretions would undergo an ecological adaptation, although lab experiments are required to prove the causal link between environmental factors and CA abundance, as well as the effects of different CA amounts in the secretions.

As we mentioned, the samples of FG secretions used in this study were 10 years old on average, some of them even 18 years old (Table 1). However, the proteinaceous content has been already proved to be stable enough to allow electrophoretic and mass spectrometry analysis (Mangiacotti et al. 2021; Mangiacotti et al. 2023a, 2023b; Mangiacotti et al. 2023). We now establish that secreted CA keeps its enzymatic activity even after 18 years from collection (lanes 4 and 7 in Figure 2 correspond to the 18‐year‐old samples), and despite the repeated freeze–thaw cycles and chemical treatments endured in previous analyses (Martín et al. 2015, 2017; Baeckens et al. 2018; Mangiacotti et al. 2021; Mangiacotti et al. 2023). Hence, femoral gland CA seems remarkably stable and possibly able to work in hard conditions, which may be expected considering the environmental stressors acting on the secretions once released on the substrate (direct solar radiation, potentially high temperature, humidity, rain, wind; Martín and López 2013; Martín et al. 2024). Experimental tests on CA‐IV from bovine red blood cells showed that the enzymatic activity drops above 65°C (Demir et al. 2000; Sharma et al. 2023), which corresponds to the denaturation temperature (Sarraf et al. 2004; Alaei et al. 2012), but it is still detectable at 60°C (Demir et al. 2000) and can be partially recovered after incubation at 70°C (Alaei et al. 2012). In different habitats occupied by Mediterranean lizards, the operative environmental temperatures measured in the field have peaks even above the limits tested for CA activity (Díaz 1997; Ortega and Pérez‐Mellado 2016; Llanos‐Garrido et al. 2023; Reppa et al. 2023). This suggests that CA in the scent marks produced by lizards with their FG secretions should be stable enough to continue playing its role even under these conditions, or at least to recover from such thermal extremes. The same rationale applies to the other enzymes occurring in the secretions (e.g., cathepsin D, phospholipase, disulfide isomerase; Tellkamp et al. 2020; Ibáñez et al. 2022; Mangiacotti et al. 2023a).

In conclusion, by showing the active state of CA in FG secretions and by relating its abundance to environmental factors, we provide preliminary support to the homeostatic function hypothesis played by (some) proteins: these proteins could actually have the potential to confer dynamic properties to the mixture, making it able to independently react to micro‐environmental changes, thus improving signal efficiency.

## Author Contributions

**Marco Mangiacotti:** conceptualization (lead), formal analysis (lead), investigation (equal), project administration (lead), software (lead), visualization (lead), writing – original draft (lead), writing – review and editing (lead). **Marco Fumagalli:** conceptualization (equal), investigation (lead), methodology (equal), writing – review and editing (equal). **Gregorio Moreno‐Rueda:** investigation (lead), writing – review and editing (equal). **Francisco J. Zamora‐Camacho:** investigation (equal), writing – review and editing (equal). **José Martìn:** investigation (equal), writing – review and editing (equal). **Roberto Sacchi:** conceptualization (equal), resources (lead), writing – review and editing (equal).

## Conflicts of Interest

The authors declare no conflicts of interest.

## Supporting information


**Figure S1:** Steps used for the quantification of Carbonic Anhydrase (CA) using SDS‐PAGE.
**Figure S2:** SDS‐PAGE gel of the FG secretion samples used for mass‐spectrometry (MS) identification of CA bands.
**Figure S3:**. Original image of the SDS‐PAGE (top) and Protonography (10 and 20 s incubation time, mid and bottom) of *Podarcis* samples used in Figure 2 of the main manuscript.
**Figure S4:**. Original image of the SDS‐PAGE (top) and Protonography (20 s incubation time; bottom) of *Psammodromus algirus* samples used in Figure 3.
**Table S1:** Pearson's correlation matrix and Variable Inflation Factors (VIF) of the bioclimatic variables used in the environmental gradient analysis.
**Table S2:** Mass Spectrometry results for the putative CA bands excised from *Psammodromus algirus* electrophoretic run.
**Table S3:** Coefficient estimates for the two discarded models (*bioclimatic1* and *topographic*) of the environmental gradient analysis.

## Data Availability

The raw data associated with this manuscript are available at zenodo.org, https://doi.org/10.5281/zenodo.15690327.
