# Supplementary material for: The Secret of Secrets: Carbonic Anhydrase Concentration in Lizards' Femoral Gland Secretions Is Tuned to Environmental Conditions
Source: Ecol Evol. 2025 Aug 21;15(8):e72023. doi: 10.1002/ece3.72023 (PMC12370373; doi:10.1002/ece3.72023)
Supplement: Supplementary file 1 — Figure S1: Steps used for the quantification of Carbonic Anhydrase (CA) using SDS‐PAGE. Figure S2: SDS‐PAGE gel of the FG secretion samples used for mass‐spectrometry (MS) identification of CA bands. Figure S3:. Original image of the SDS‐PAGE (top) and Protonography (10 and 20 s incubation time, mid and bottom) of Podarcis samples used in Figure 2 of the main manuscript. Figure S4:. Original image of the SDS‐PAGE (top) and Protonography (20 s incubation time; bottom) of Psammodromus algirus samples used in Figure 3. Table S1: Pearson's correlation matrix and Variable Inflation Factors (VIF) of the bioclimatic variables used in the environmental gradient analysis. Table S2: Mass Spectrometry results for the putative CA bands excised from Psammodromus algirus electrophoretic run. Table S3: Coefficient estimates for the two discarded models (bioclimatic1 and topographic) of the environmental gradient analysis. [file ECE3-15-e72023-s001.pdf]

The secret of secrets: carbonic anhydrase concentration in lizards' femoral gland secretions is tuned to environmental conditions

MARCO MANGIACOTTI[1,*], MARCO FUMAGALLI[1], GREGORIO MORENO-RUEDA[2], FRANCISCO J. ZAMORA-CAMACHO[2,3], JOSÉ MARTÌN[4], ROBERTO SACCHI[1]

1 Department of Earth and Environmental Sciences, University of Pavia, Università degli Studi di Pavia, via Taramelli 24, IT27100 Pavia.
2 Departamento de Zoología, Facultad de Ciencias, Universidad de Granada, 18071 Granada, Spain
3 Departamento de Biología de Organismos y Sistemas, Universidad de Oviedo, 33071 Oviedo, Spain
4 Departamento de Ecología Evolutiva, Museo Nacional de Ciencias Naturales, CSIC, José Gutiérrez Abascal 2, E-28006 Madrid, Spain
**Corresponding author*. Email: marco.mangiacotti@unipv.it

SUPPLEMENTARY MATERIALS

Fig. S1. Steps used for the quantification of Carbonic Anhydrase (CA) using SDS-PAGE. A) Example of an acquired SDS-PAGE gel image including six different samples (lanes 2-7). B) Zoom of lane six and associated normalized electrophoretic profile. The arrow points the position of the CA band; red colour marks the area under CA peak used as a proxy for CA concentration.

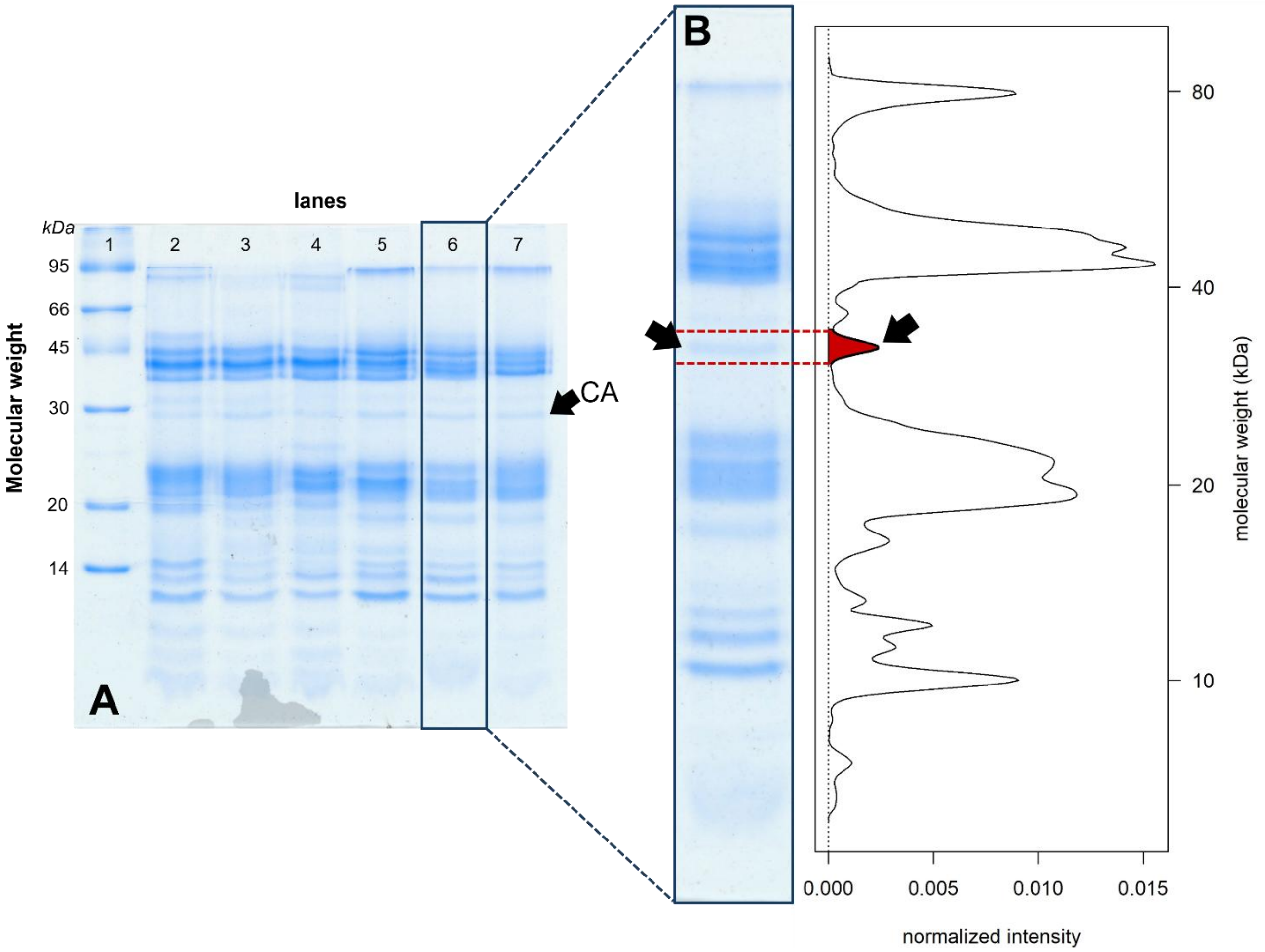

Fig S2. SDS-PAGE gel of the FG secretion samples used for mass-spectrometry (MS) identification of CA bands. In red boxes, the three excised bands sent to MS and coming from samples of three different lizards. Lanes 2 and 4 came from the same individual but with different protein amount loaded (about 5 µg in lane 2, about 10 µg in the other lanes), to check the best protein amount for MS. Molecular weights ran in lanes 1 and 10.

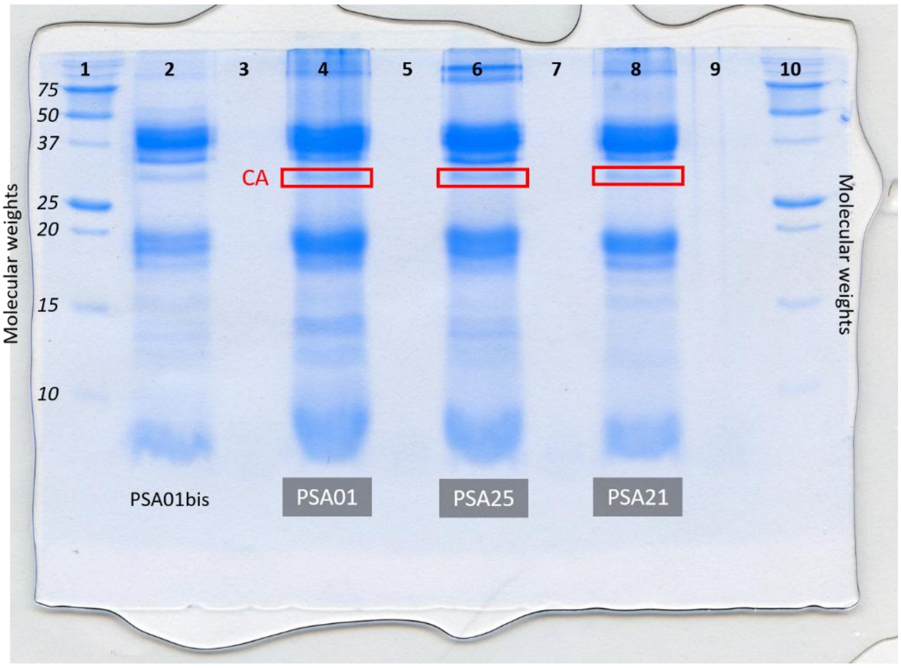

Fig. S3. Original image of the SDS-PAGE (top) and Protonography (10 and 20 s incubation time, mid and bottom) of *Podarcis* samples used in Fig. 2 of the main manuscript. The sample order is the same as in Fig. 2. An additional lane (last on the right side of each gel) was omitted from Fig. 2 since it was loaded with a fresh sample of *Podarcis muralis* (collected in Pavia on May 2024) and used only as a control for any potential lack of detection in protonography due to the age of the samples.

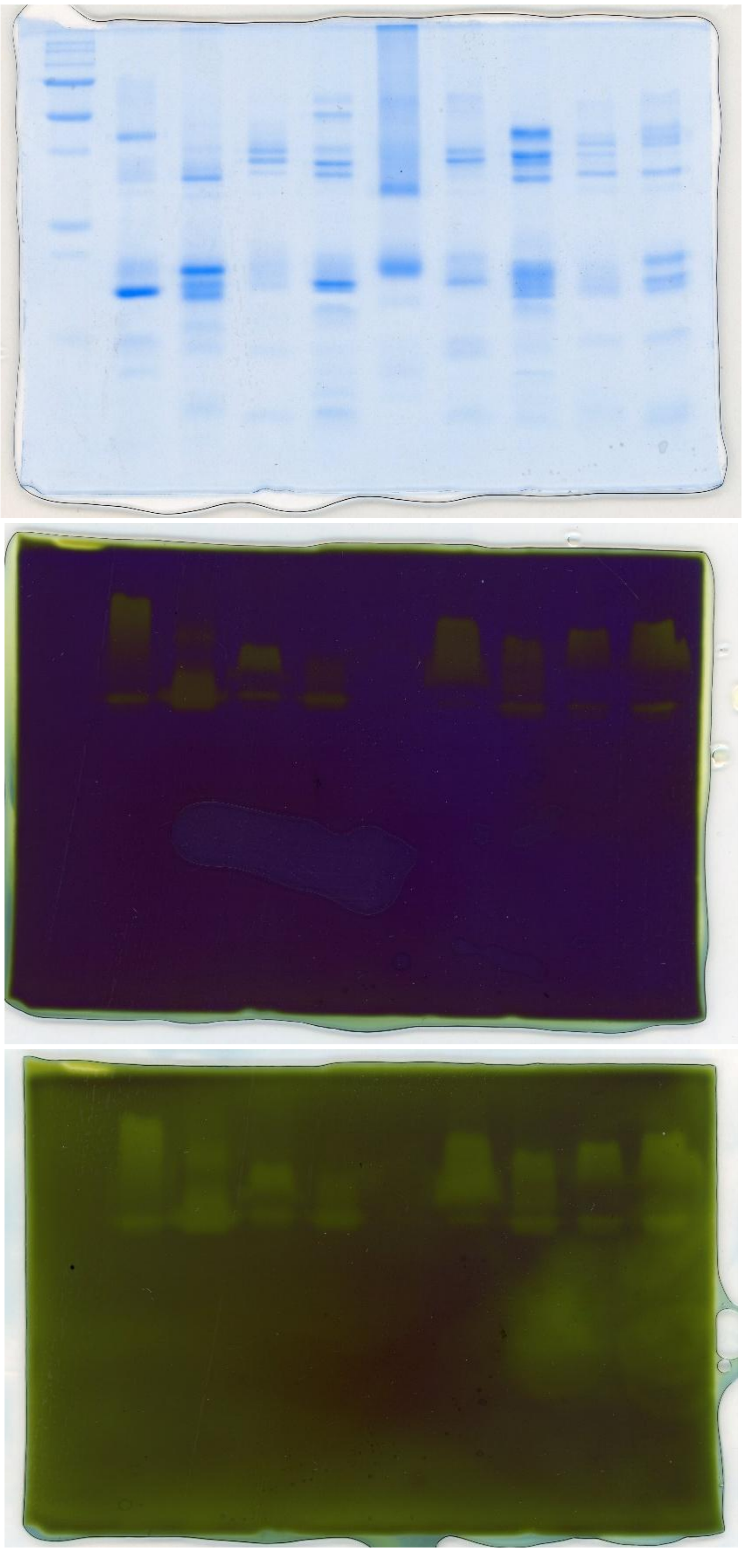

Fig. S4. Original image of the SDS-PAGE (top) and Protonography (20 s incubation time; bottom) of *Psammodromus algirus* samples used in Fig. 3 of the main manuscript. The sample order is the same as in Fig. 3.

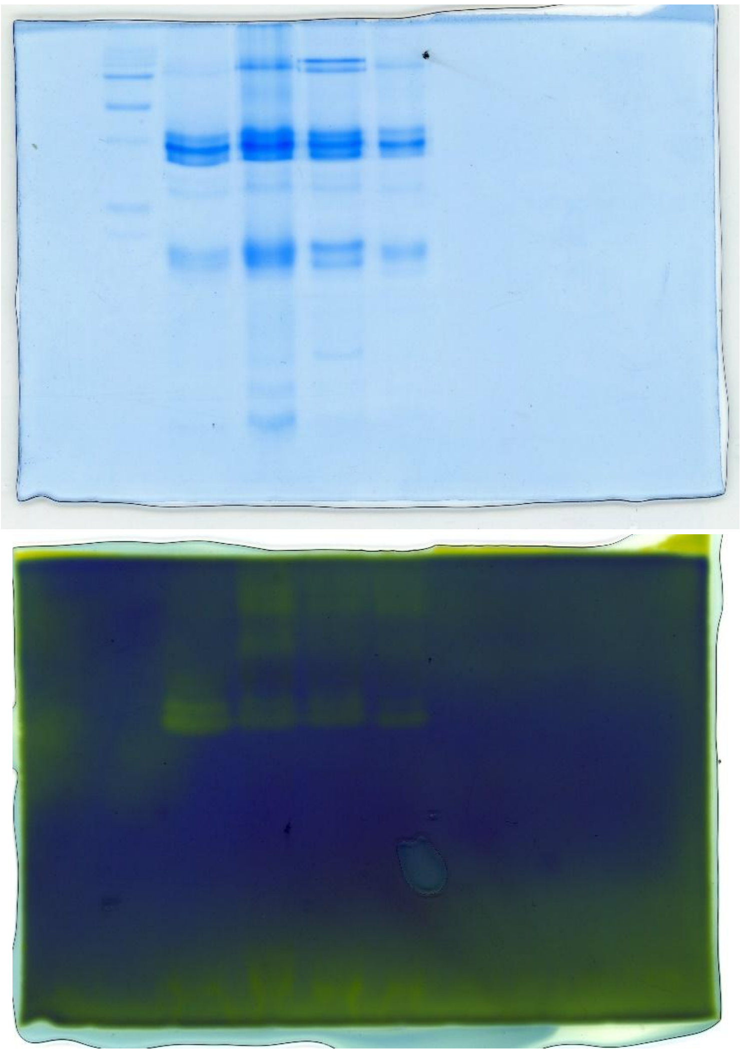

Tab. S1. Pearson's correlation matrix and Variable Inflation Factors (VIF) of the bioclimatic variables used in the environmental gradient analysis. VIF values are reported considering: the full set of predictors (*All*); the two subsets of bioclimatic variables used as predictors in the two bioclimatic models (*bioc1* and *bioc2*). Altitude = elevation a.s.l.; T_{avg} = average annual temperature; T_{range} = temperature mean diurnal range; $Prec_{annual}$ = total annual precipitation; $Prec_{CV}$ = Precipitation seasonality (coefficient of variation).

	**Pearson correlation coefficient**				**VIF**		
	T_{avg}	T_{range}	$Prec_{annual}$	$Prec_{CV}$	*All*	*Bioc1*	*Bioc2*
**Altitude**	-0.897	0.656	0.949	-0.368	—	—	—
**T_{avg}**	—	-0.529	-0.903	0.671	23.296	2.388	—
**T_{range}**	—	—	0.682	-0.268	2.546	1.414	1.870
**$Prec_{annual}$**	—	—	—	-0.379	19.766	—	2.027
**$Prec_{CV}$**	—	—	—	—	4.641	1.855	1.168

Tab. S2. Mass Spectrometry results for the putative CA bands excised from *Psammodromus algirus* electrophoretic run. Sample = individual lizard used; db = database against which peptide search was done (CAvert = UniprotKB available sequence matching query for carbonic anhydrase and filtered for vertebrates; Podlil = *Podarcis lilfordi* proteome; Podmur = *P. muralis* proteome); Accession= uniprotKB accession; Description = uniprot protein description. Error = difference between the measured and calculated parental ion mass (Da); Score = MSGF+ spectrum E-value (-log_{10} transformed). Green-shaded rows correspond to high-score, low error identified peptides, which clearly outperformed other potential matches.

Sample	db	Accession	Description	Peptide	Error	Score
**PSA01**	CAvert	A0AA35PK31	Carbonic anhydrase	K.FTSSLFFTTK.Q	0.001	13.173
		A0AA35PK31	Carbonic anhydrase	K.GDYPDVEMK.E	0.004	12.593
		H3BZ23	Protein tyrosine phosphatase receptor type Z1a	R.SSSVLADYALR.T	1.005	6.639
		A0A8J4U8E4	Carbonic anhydrase 12 isoform X1	K.AVFPQLNLK.S	-0.029	6.339
		A0A9L0RAC2	Carbonic anhydrase	R.LHSFFLSFHK.H	-0.021	5.680
		A0A7K9RTN9	Carbonic anhydrase (Fragment)	R.VLSPAQVR.A	1.024	5.330
		A0A8C8K7C2	protein-tyrosine-phosphatase	R.VSLELGSALQPR.R	-0.006	5.320
		A0A6F9CKN3	Alpha-carbonic anhydrase domain-containing protein (Fragment)	K.ELHIVNIK.E	0.005	5.190
		A0A7K6AFV8	Carbonic anhydrase 4 (Fragment)	K.NSTHMADNFRPAQLLYER.K	0.009	5.069
	Podlil	A0AA35PK31	Carbonic anhydrase	K.FTSSLFFTTK.Q	0.001	13.221
		A0AA35PK31	Carbonic anhydrase	K.GDYPDVEMK.E	0.004	11.834
		A0AA35KX53	Bromodomain-containing protein 1 isoform X1	R.IFAQPVNLK.E	-0.029	7.491
		A0AA35KTV0	LRAT domain-containing protein	K.FLLLANFK.L	0.000	6.248
	Podmur	A0A670JGA4	Carbonic anhydrase	K.GDYPDVEMK.E	0.004	12.125
		A0A670J7Y2	Bromodomain containing 1	R.IFAQPVNLK.E	-0.029	7.504
		A0A670KMG8	Reverse transcriptase domain-containing protein	R.LLTGSLPR.E	0.004	6.641
		A0A670I4M0	Lysosomal trafficking regulator	R.SVITPLLHSFR.S	-0.021	6.273
		A0A670J3G0	Lecithin retinol acyltransferase	K.FLLLANFK.L	0.000	6.260
**PSA21**	CAvert	A0A6J0UUN5	Carbonic anhydrase	R.YAMELHIVHTK.N	0.004	15.040
		A0AA35PK31	Carbonic anhydrase	K.FTSSLFFTTK.Q	0.004	13.113
		H3BZ23	Protein tyrosine phosphatase receptor type Z1a	R.SSSVLADYALR.T	1.003	6.671
		A0A665ULT1	protein-tyrosine-phosphatase	K.FLLPWPEK.H	0.000	6.631
		A0A8C9BSX1	Carbonic anhydrase	R.DGLAVLAALVEVK.D	-0.032	6.308
		A0A9L0RAC2	Carbonic anhydrase	R.LHSFFLSFHK.H	-0.023	5.885
		A0A803SNU6	protein-tyrosine-phosphatase	R.EDDAHGK.D	-0.012	4.593
	Podlil	A0AA35PK31	Carbonic anhydrase	K.FTSSLFFTTK.Q	0.004	13.277
		A0AA35L161	Endoplasmic reticulum aminopeptidase 1	K.FMEFVSVNVTHPELK.V	0.000	8.309
		A0AA35KJ38	Dihydropyrimidinase isoform X1	R.FVAVTSTNTAK.I	-0.029	6.971
		A0AA35PH18	Ectopic P-granules autophagy protein 5 homolog	K.LFLWWHK.I	0.010	6.925
		A0AA35KTV0	LRAT domain-containing protein	K.FLLLANFK.L	-0.001	6.689

	Podmur	A0A670K7I3	Dihydropyrimidinase	R.FVAVTSTNTAK.I	-0.029	7.047
		A0A670JHC8	Ectopic P-granules autophagy protein 5 homolog	K.LFLWWHK.I	0.010	6.923
		A0A670JSR8	NADH dehydrogenase [ubiquinone] iron-sulfur protein 2, mitochondrial	R.QGIALGAAPSR.G	0.027	6.884
		A0A670J3G0	Lecithin retinol acyltransferase	K.FLLLANFK.L	-0.001	6.672
		A0A670KMG8	Reverse transcriptase domain-containing protein	R.LLTGSLPR.E	0.001	6.425
		A0A670I5J7	Epithelial cell transforming 2 like	R.FTELLEDALK.A	-0.017	6.388
		A0A670JQ46	dual-specificity kinase	R.GAAAAAK.T	1.001	3.437
**PSA25**	CAvert	A0AA35PK31	Carbonic anhydrase	K.FTSSLFFTTK.Q	0.004	13.077
		A0AA35PK31	Carbonic anhydrase	K.GDYPDVEMK.E	0.002	12.684
		H3BZ23	Protein tyrosine phosphatase receptor type Z1a	R.SSSVLADYALR.T	1.004	6.692
		A0A665ULT1	protein-tyrosine-phosphatase	K.FLLPWPEK.H	0.002	6.609
		A0A673AMF9	Alpha-carbonic anhydrase domain-containing protein	R.DSTGLAALGFLIDVR.N	0.995	6.423
		A0A8C9BSX1	Carbonic anhydrase	R.DGLAVLAALVEVK.D	-0.030	6.028
		G1KFX3	protein-tyrosine-phosphatase	K.YSDQVIVDMPLDDPGK.F	1.048	5.646
		A0A673UZT3	Alpha-carbonic anhydrase domain-containing protein	R.GTCSGAAPQR.S	0.999	5.589
		A0A9L0RAC2	Carbonic anhydrase	R.LHSFFLSFHK.H	-0.022	5.499
		A0A8C8K7C2	protein-tyrosine-phosphatase	R.VSLELGSALQPR.R	-0.006	5.498
		A0A6P9CGQ1	Carbonic anhydrase	R.DLIHLSK.K	0.999	5.230
		Q4SE53	Chromosome undetermined SCAF14625, whole genome shotgun sequence (Fragment)	R.TGLAVSVSDR.D	-0.024	5.230
		G1KFX3	protein-tyrosine-phosphatase	R.EDDAHGK.D	-0.012	4.652
	Podlil	A0AA35PK31	Carbonic anhydrase	K.FTSSLFFTTK.Q	0.004	13.338
		A0AA35PK31	Carbonic anhydrase	K.GDYPDVEMK.E	0.002	12.105
		A0AA35L161	Endoplasmic reticulum aminopeptidase 1	K.FMEFVSVNVTHPELK.V	0.003	7.461
		A0AA35KDK0	Cation-transporting ATPase	K.DGYDLYMK.G	1.017	7.356
		A0AA35PH18	Ectopic P-granules autophagy protein 5 homolog	K.LFLWWHK.I	0.011	7.271
		A0AA35JTD2	DUF4392 domain-containing protein	K.CPSLSSDADIR.T	0.009	5.864
	Podmur	A0A670JGA4	Carbonic anhydrase	K.GDYPDVEMK.E	0.002	12.318
		A0A670KH59	Cation-transporting ATPase	K.DGYDLYMK.G	1.017	7.501
		A0A670JHC8	Ectopic P-granules autophagy protein 5 homolog	K.LFLWWHK.I	0.011	7.292
		A0A670HQN6	Aladin WD repeat nucleoporin	K.TASIVADLSETTFETLYGEER.I	0.968	6.284
		A0A670JBC0	G-protein coupled receptors family 3 profile domain-containing protein	R.MLPNVSLGFHIYDNYYDSK.M	-0.003	5.983
		A0A670HM82	D-glutamate cyclase	K.CPSLSSDADIR.T	0.009	5.821
		A0A670JQ46	dual-specificity kinase	R.GAAAAAK.T	1.001	3.680

Table S3. Coefficient estimates for the two discarded models (*bioclimatic1* and *topographic*; Tab. 2 and Fig. 4A of the main text) of the environmental gradient analysis. T_{avg} = average annual temperature; T_{range} = mean diurnal temperature range; $Prec_{CV}$ = precipitation seasonality; altitude = elevation a.s.l.; Parameter = predictor for which parameter was estimated; MAP = Maximum A Posteriori probability estimate; HDI_{95} = 95% high density interval; MPE = Maximum Probability of Effect, probability of the parameter being strictly positive or negative.

**model**	**Parameter**	**MAP**	**HDI_{95}**		**MPE**
			Lower	*Upper*	
bioclimatic1	Intercept	1.935	1.755	2.108	1.000
	T_{avg}	-0.147	-0.468	0.112	0.896
	T_{range}	0.037	-0.181	0.276	0.681
	$Prec_{CV}$	0.304	0.089	0.561	0.985
topographic	Intercept	1.954	1.757	2.167	1.000
	Altitude	0.077	-0.131	0.275	0.744
